# Local imaging to interpret tumor size in F18 fluorodeoxyglucose positron emission tomography/CT in lung cancers

**DOI:** 10.1590/1806-9282.20230762

**Published:** 2024-03-04

**Authors:** Şadiye Altun Tuzcu, İhsan Kaplan, İbrahim İbiloğlu, Ali Uyar, Fatih Güzel, Yunus Güzel, Bekir Taşdemir

**Affiliations:** 1 Dicle University Medical Faculty, Department of Nuclear Medicine – Diyarbakır, Turkey.; 2 Diyarbakır Gazi Yaşargil Education and Research Hospital, Department of Nuclear Medicine – Diyarbakır, Turkey.; 3 Dicle University Medical Faculty, Department of Pathology – Diyarbakır, Turkey.; 4 Bilecik Research and Education Hospital, Department of Nuclear Medicine – Bilecik, Turkey.

**Keywords:** Thorax, Whole body imaging, Solitary pulmonary nodule, Lung, Cancer

## Abstract

**OBJECTIVE:**

This study aimed to determine the thoracic and extra-thoracic extension of the disease in patients diagnosed with lung cancer and who had whole-body F18-fluorodeoxyglucose positron emission tomography/CT imaging and to investigate whether there is a relationship between tumor size and extrathoracic spread.

**METHODS:**

A total of 308 patients diagnosed with lung cancer were included in this study. These 308 patients were first classified as group 1 (SPN 30 mm>longest lesion diameter ≥10 mm) and group 2 (lung mass (longest lesion diameter ≥30 mm), and then the same patients were classified as group 3 (nodular diameter of ≤20 mm) and group 4 (nodular size of >20 mm). Group 1 was compared with group 2 in terms of extrathoracic metastases. Similarly, group 3 was compared with group 4 in terms of frequency of extrathoracic metastases. F18 fluorodeoxyglucose positron emission tomography/CT examination was used to detect liver, adrenal, bone, and supraclavicular lymph node metastasis, besides extrathoracic metastasis.

**RESULTS:**

Liver, bone, and extrathoracic metastasis in group 1 was statistically lower than in group 2 (p<0.001, p<0.01, and p=0.03, respectively). Liver, extrathoracic, adrenal, and bone metastasis in group 3 was statistically lower than that in group 4 (p<0.001, p=0.01, and p=0.04, p<0.01, respectively). The extrathoracic extension was observed in only one patient in group 3. In addition, liver, adrenal, and bone metastases were not observed in group 3 patients.

**CONCLUSION:**

Positron emission tomography/CT may be more appropriate for cases with a nodule diameter of ≤20 mm. Performing local imaging in patients with a nodule diameter of ≤20 mm could reduce radiation exposure and save radiopharmaceuticals used in positron emission tomography/CT imaging.

## INTRODUCTION

The 18F-FDG (fluorodeoxyglucose) PET (positron emission tomography) is a sensitive imaging method used to diagnose and evaluate staging, restaging, and treatment response in oncology. Anatomical and morphological information obtained by CT can improve the localization, extent, and characterization of lesions detected by FDG PET^1^.

The evaluation of solitary lung nodules via FDG PET/CT has high sensitivity for nodules larger than 8 mm and is utilized to exclude lung cancer^2-6^. The positivity of PET is elaborated by the visualization of FDG uptake in the nodule higher than the mediastinal blood flow^3^. However, applying nonsurgical treatments in patients with PET-positive lesions requires histopathological confirmation^2-5^. The nodules that do not retain FDG are considered benign and the transthoracic biopsy process can be avoided in these patients^5^.

The sensitivity of FDG PET/CT imaging is limited to a small size (diameter less than 1 cm) and nonsolid nodules. These nodules should be followed up with a structured framework of CT^6^. The false negative outcomes of FDG PET/CT imaging are often associated with nonsolid parenchymal nodules and adenocarcinoma in situ, a subtype of adenocarcinomas (formerly known as bronchoalveolar carcinoma)^7^. FDG PET/CT imaging in situ adenocarcinoma sensitivity is 33–38% in ground-glass density lung nodules^8^. FDG PET/CT presents relatively low sensitivity (75%) in carcinoid lung tumors^8,9^. When small size and nonsolid lung nodules are excluded, the sensitivity of FDG PET/CT imaging ranges from 88 to 100% in the diagnosis of lung cancer^10^.

Statements that PET/CT applications cause high radiation exposure have increased recently. The CT component accounts for over 50% of the total radiation emission^10,11^. An effective dose such as 14 mSv administered with PET/CT increased the risk of cancer due to radiation by 0.07–0.62%^12^.

The present study aimed to investigate whether it was sufficient to include only a local area in F18 FDG PET/CT imaging, considering the diameter of the lung lesion, which is applied for staging purposes in patients with pre-diagnosis and lung cancer diagnosis.

## METHODS

Of the 308 patients who applied for FDG PET/CT imaging, 44 were diagnosed with small-cell lung cancer, 238 with non-small-cell lung cancer, 17 with a neuroendocrine tumor, and 9 with sarcomatoid carcinoma. Metastatic lesions were confirmed histopathologically or in clinical and/or radiological follow-up. The study was conducted in accordance with the Declaration of Helsinki and followed ethical standards. The ethics committee approved the study in 2020, and the protocol number is 141.

According to the size of the longest diameter of the lesion, the patients were first classified into groups 1 and 2. Group 1 lesion included 74 patients (mean age 62.7±14.5 years) with the longest diameter of 10 mm or greater than 10 mm and less than 30 mm, and group 2 included 234 patients (mean age 63.7±11.6 years) with lesions of 30 mm and above in the longest diameter of the lesion.

The same patients were classified into two more groups: groups 3 and 4. Group 3 patients comprised 32 patients (mean age 64.1±14.7 years) with a diameter less than or equal to 20 mm, and group 4 consisted of 276 patients (mean age 63.4±12.0 years) larger than 20 mm. Group 1 was compared with group 2, and group 3 was compared with group 4.

Fluorodeoxyglucose PET/CT imaging was performed in all patients to evaluate the presence of thoracic metastasis (lung, soft-tissue organ, bone, and lymph node metastasis in the region, including supraclavicular lymph node in the upper limit and liver-adrenal organs in the lower limit) or extrathoracic metastasis (soft-tissue organ, bone, and lymph node metastasis other than in the defined thoracic region).

### Statistical analysis

The IBM SPSS 21.0 for Windows statistical package program was used for the statistical evaluation of the data. Measurable variables were presented as mean±standard deviation and categorical variables as numbers and percentages (%). The chi-square (^2^) test was used for comparing categorical variables, and the independent-sample t-test was used for comparing measurable variables. A p≤0.05 indicated a statistically significant difference.

## RESULTS

The longest mean tumor diameter was determined to be 21.6±6.0 mm in group 1 and 65.8±41.9 mm in group 2. A significant difference was found between the two groups in terms of the longest tumor diameter (p<0.0001).

**Figure 1.**
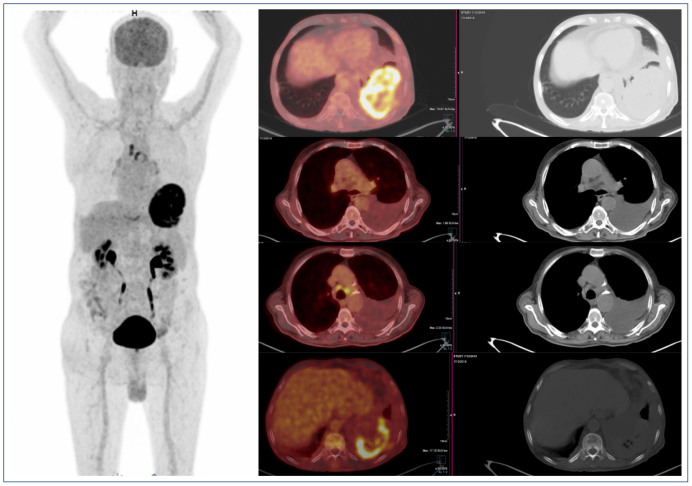
Bone and lymph node metastases in a patient with a mass of 11×7 cm in the lung.

In group 1, 61 patients (82.4%) did not have extrathoracic metastasis, and 13 (17.6%) had extrathoracic metastasis. The bone and lymph node metastasis of a single patient is elaborated in Figure 1. In group 2, 139 patients (59.4%) did not have extrathoracic metastasis, and 95 patients (40.6%) had extrathoracic metastasis. A statistically significant difference was observed in extrathoracic metastasis between the two groups (p<0.001). The findings of a 19×15 mm nodule, lymph node metastasis, and parenchymal spread in the lung of a single patient are elaborated.

In all, 70 patients in group 1 (94.6%) did not have liver metastasis, but 4 (5.4%) patients had liver metastasis. Notably, 201 (85.9%) patients in group 2 did not have liver metastasis, but 33 (14.1%) patients had liver metastasis. A statistically significant difference was found in liver metastasis between the two groups (p=0.03).

In group 1, 71 (95.9%) patients did not have adrenal metastasis, and 3 (4.1%) patients had adrenal metastasis. In contrast, 210 (89.7%) patients did not have adrenal metastasis, and 24 (10.3%) patients had adrenal metastasis in group 2.

A total of 71 (95.9%) patients did not have supraclavicular lymph node metastasis, and 3 (4.1%) patients had supraclavicular lymph node metastasis in group 1. In group 2, 213 (91%) patients did not have supraclavicular lymph node metastasis, and 21 (9%) patients had supraclavicular lymph node metastasis. Supraclavicular lymph node metastasis was statistically lower in group 1 (p<0.05).

In all, 68 (91.8%) patients did not have bone metastasis, and 6 (8.1%) patients had bone metastasis in group 1. In group 2, 192 (82%) patients did not have bone metastasis, and 42 (17.9%) patients had bone metastasis. Bone metastasis in group 1 was significantly lower than in group 2 (p<0.01) (Table 1).

**Table 1.** Comparison of SPN (nodule≤30 mm) and lung masses.

	Group 1 (n=74)	Group 2 (n=234)	p-value
Age (years)	62.7±14.5	63.7±11.6	>0.05
Sex (female/male)	25/49	27/207	<0.0001
Longest tumor diameter (mm)	21.6±6	63.7±11.6	<0.0001
Extrathoracic metastasis(yes/no)	13/61	95/139	<0.001
Liver metastasis (yes/no)	4/70	33/201	0.03
Adrenal metastasis (yes/no)	3/71	24/210	>0.05
Supraclavicular lymph node metastasis (yes/no)	3/71	21/213	>0.05
Bone metastasis (yes/no)	6/68	42/192	<0.01

Extrathoracic metastasis was determined in only 1 (3.1%) patient in group 3. Extrathoracic metastasis was found in 107 (38.8%) patients in group 4. A statistically significant difference was noted in terms of extrathoracic metastasis between group 3 and group 4 (p<0.001).

Liver metastasis was not found in group 3. Liver metastasis was found in 37 (13.4%) patients in group 4. A statistically significant difference was observed in liver metastasis between the two groups (p<0.01).

Adrenal metastasis was not found in group 3. Adrenal metastasis was found in 27 (8.8%) patients in group 4. A statistically significant difference was found in adrenal metastasis between group 3 and group 4 (p=0.044).

Supraclavicular lymph node metastasis was not found in 30 (93.8%) patients and found in 2 (6.2%) patients in group 3. Supraclavicular lymph node metastasis was not found in 254 (92%) patients in group 4. Supraclavicular lymph node metastasis was found in 22 (8%) patients in group 4 Supraclavicular lymph node metastasis in group 3 was significantly lower than that in group 4 (p<0.05).

Bone metastasis was not found in group 3. In contrast, 48 (17%) patients in group 4 had bone metastasis. Bone metastasis in group 3 was significantly less than that in group 4 (p<0.01) (Table 2).

**Table 2.** Comparison of SPN (nodule≤20 mm) and lung masses.

	Group 3 (n=32)	Group 4 (n=276)	p-value
Age (years)	64.1±14.7	63.4±12.0	>0.05
Sex (female/male)	12/20	40/236	0.001
Longest tumor diameter (mm)	15.5±3.2	59.9±41.0	0.0001
Extrathoracic metastasis (yes/no)	1/31	107/169	<0.001
Liver metastasis (yes/no)	0/32	37/239	0.01
Adrenal metastasis (yes/no)	0/32	27/249	0.04
Supraclavicular lymph node metastasis (yes/no)	2/30	22/254	>0.05
Bone metastasis (yes/no)	0/32	48/228	<0.01

## DISCUSSION

Thorax constitutes approximately half of the body on a conventional PET/CT scan. A segmental scan can obtain a sufficient count rate by administering half or less of the standard FDG dose. Alternatively, the scan time can be reduced by up to two-thirds by keeping the dose constant. In case of need, a whole-body scan can be used to complete the study^13,14^. Limiting the imaging field and reducing the FDG dose can save 4.45–9.1 mSv for each procedure^9,15^. A reduction in intake time and the amount of FDG applied can significantly improve workflow and lab productivity. By shortening the scanning time, time can be created for respiratory gating and dual-phase imaging, in turn improving diagnostic accuracy. Local imaging can improve the balance between the cost and effectiveness of PET/CT scans in patients with SPN^16-18^.

Although potential advantages are associated with adopting a segmental PET/CT scan strategy, some critical issues should be considered. In the present study, significantly less extrathoracic metastasis was detected in patients with solitary pulmonary nodules than in those with lung masses. While approximately 1 in 6 (17.6%) patients with SPN having a nodule diameter of ≤30 mm had extrathoracic metastasis, 2 of 5 (40.6%) patients with lung masses showed distant metastasis. Although liver metastasis was significantly lower in patients with SPN than those with lung masses, no difference was found in adrenal metastasis. Extrathoracic metastasis was significantly lower in patients with SPN with a nodule diameter of ≤20 mm than in those with lung masses. These results indicated that local imaging might be an appropriate approach in patients with a nodule diameter of ≤20 mm compared with patients with a nodule diameter of ≤30 mm. When the nodule diameter is <30 mm, 17% of patients with extrathoracic metastasis are missed, while only 3% of patients with a nodule diameter <20 mm are missed by local imaging. If local imaging is to be performed, it may be a more appropriate approach for patients with a nodule diameter of <20 mm^19^. Osman et al., reported that whole-body imaging caused a change in treatment in 2.6% of 500 patients and that whole-body imaging was necessary^20^.

A study conducted with more than 300 patients with SPN in Italy reported that extrathoracic metastasis was observed in 5% of the patients and that only 2% were suspected of having lung cancer metastasis. In the same study, the researchers stated that the segmental approach was 6 min and 3 s shorter per scan compared with the standard PET/CT scan and that imaging only the thorax would reduce the FDG dose by approximately 60% compared with whole-body imaging^21^. Similarly, the present study found that only 3.1% of patients with SPN, especially those with a nodule diameter of ≤20 mm, had extrathoracic metastasis. Spadafora et al.^21^ recommended that local imaging in especially limited patient groups would reduce the cost, time, and radiation dose that patients would be exposed to, similar to the present study.

## CONCLUSION

Considering the demand for health care services and the increase in daily costs, local imaging seems appropriate, especially in SPN patients with mass diameters. If this innovative procedure can be adopted, it may bring many benefits to both physicians and patients. Scanning only the thorax, especially in selected patients with SPN, may reduce the risk of radiation exposure and costs as the thorax occupies about half of the body. Investigating this issue with larger patient groups may shed more light.

## INFORMED CONSENT

Informed consent was obtained from all the patients before the initiation of the study.

## INSTITUTIONAL REVIEW BOARD APPROVAL

The ethics committee approval date is 2020, and the protocol number is 141.
